# Characterization of Bacteria in Biopsies of Colon and Stools by High Throughput Sequencing of the V2 Region of Bacterial 16S rRNA Gene in Human

**DOI:** 10.1371/journal.pone.0016952

**Published:** 2011-02-10

**Authors:** Yukihide Momozawa, Valérie Deffontaine, Edouard Louis, Juan F. Medrano

**Affiliations:** 1 Unit of Animal Genomics, GIGA-Research and Faculty of Veterinary Medicine, University of Liège, Liège, Belgium; 2 Unit of Hepatology and Gastroenterology, Department of Clinical Sciences, GIGA-Research and Faculty of Medicine, and CHU de Liège, University of Liège, Liège, Belgium; 3 Department of Animal Science, University of California Davis, Davis, California, United States of America; University of Birmingham, United Kingdom

## Abstract

**Background:**

The characterization of the human intestinal microflora and their interactions with the host have been identified as key components in the study of intestinal disorders such as inflammatory bowel diseases. High-throughput sequencing has enabled culture-independent studies to deeply analyze bacteria in the gut. It is possible with this technology to systematically analyze links between microbes and the genetic constitution of the host, such as DNA polymorphisms and methylation, and gene expression.

**Methods and Findings:**

In this study the V2 region of the bacterial 16S ribosomal RNA (rRNA) gene using 454 pyrosequencing from seven anatomic regions of human colon and two types of stool specimens were analyzed. The study examined the number of reads needed to ascertain differences between samples, the effect of DNA extraction procedures and PCR reproducibility, and differences between biopsies and stools in order to design a large scale systematic analysis of gut microbes. It was shown (1) that sequence coverage lower than 1,000 reads influenced quantitative and qualitative differences between samples measured by UniFrac distances. Distances between samples became stable after 1,000 reads. (2) Difference of extracted bacteria was observed between the two DNA extraction methods. In particular, *Firmicutes Bacilli* were not extracted well by one method. (3) Quantitative and qualitative difference in bacteria from ileum to rectum colon were not observed, but there was a significant positive trend between distances within colon and quantitative differences. Between sample type, biopsies or stools, quantitative and qualitative differences were observed.

**Conclusions:**

Results of human colonic bacteria analyzed using high-throughput sequencing were highly dependent on the experimental design, especially the number of sequence reads, DNA extraction method, and sample type.

## Introduction

High-throughput sequencing has significantly accelerated the characterization of human bacteria in several phenotypes and diseases by metagenomic [1] and 16S rRNA-based techniques [2]. In human colon, the proportions of phylum *Firmicutes* and class *Clostridia* was found to be significantly reduced in diabetics [3]. Obesity was also associated with reducing bacterial diversity and altering representation of bacterial genes and metabolic pathways [4]. While these reports provided new insights, a wide range of approaches will be needed to reveal the role of specific bacteria in relation to phenotypes and diseases [2]. One approach is a systematic analysis using high-throughput methods to examine links between bacteria and host genetic factors, such as DNA polymorphisms, DNA methylation, and gene expression. However, more information is needed to design a systematic sampling and analysis approach using high-throughput methods.

High-throughput sequencing provides a high number of reads at a relatively low cost, but researchers still must decide upon a reasonable number of reads to ascertain associations between samples and to detect rare bacteria. This clearly depends on the purpose of the research. Fewer than 100 reads are enough to determine the ratio between two dominant phyla (*Bacteroidetes* and *Firmicutes*) and to detect the major patterns of variation among the microbial communities in the guts of diverse mammals [5]. However, it is not yet clear how many reads would be needed to ascertain differences between individuals and between anatomic regions of the same individual.

Most studies examining colon bacteria have used stool samples. These samples have worked well as proxy to reveal interindividual differences in several studies (e.g. [6], [7]). However, bacteria in stool and biopsy samples of the same individual have been reported to be different by Sanger sequencing colonies of amplicons, although these differences were smaller than differences between individuals [8]. It needs to be determined how bacteria of biopsies in colon and stools are different by deeper sequencing to find out if stools effectively work as proxy. In addition, the difference between anatomic regions in colon should be determined to decide upon target regions for sampling.

In this study, the V2 region of the bacterial 16S ribosomal RNA (rRNA) from seven anatomic colon regions and two sampling types of stools were analyzed to determine: (1) The number of reads to ascertain differences between samples (2) the effect of different DNA extraction procedures and PCR reproducibility and (3) differences in bacterial content between seven anatomic regions and two sampling types of stools.

## Results

### Coverage and UniFrac distances

**Figure 1:**
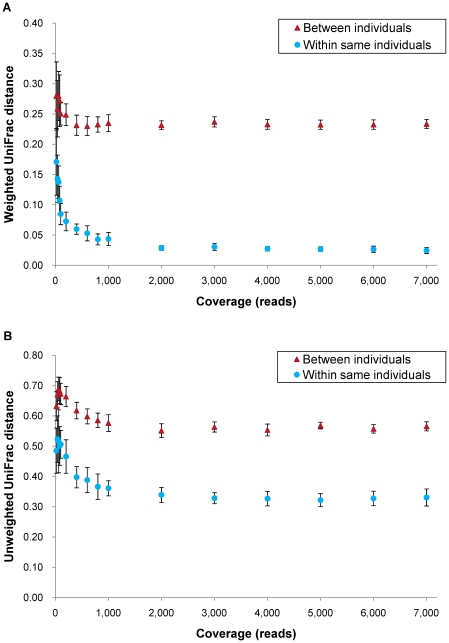
Association between coverage and UniFrac distances at different coverage. (A) Weighted and (B) unweighted UniFrac distances. For ‘Between individuals’ each data point includes 36 distances calculated from 6 samples of individual B and 6 samples of individual C. For ‘Within same individual’ each data point includes 18 distances (9 within sample B and 9 within sample C). Mean ± SD.

Figures 1A and 1B show the association between coverage and UniFrac distances (weighted and unweighted), respectively. Values were estimated by randomly sampling different number of reads from two PCR and deep sequencing runs in each DNA of the two individuals. Both distances between individuals were higher than those between the same individual at any coverage, with weighted and unweighted distances showing a similar tendency. While the distances decreased according to coverage between 0 and 1,000 reads, they became stable after 1,000 reads. Weighted distance, accounting for abundance of operational taxonomic units (OTUs), reached a plateau at 0.025 within the same individual and 0.23 between different individuals. Unweighted distance, accounting for only presence or absence of OTUs, reached plateaus at 0.32 and 0.55, respectively.

### Influence of chimeric sequences

To investigate influence of chimeric sequences produced during PCR, we performed the above analysis with and without elimination of definite chimeric sequences with B2C2 software [9] to compare the average values at each coverage. B2C2 detected 1.21% and 5.52% as definite chimeric sequences in individuals B and C, respectively. A small but significant decrease in unweighted distances between individuals was observed (mean ± SD: −0.015±0.011, t_15_ = 5.79, p = 3.58×10^-5^) after correction for chimeric sequences.

### Coverage and the number of OTUs

**Figure 2:**
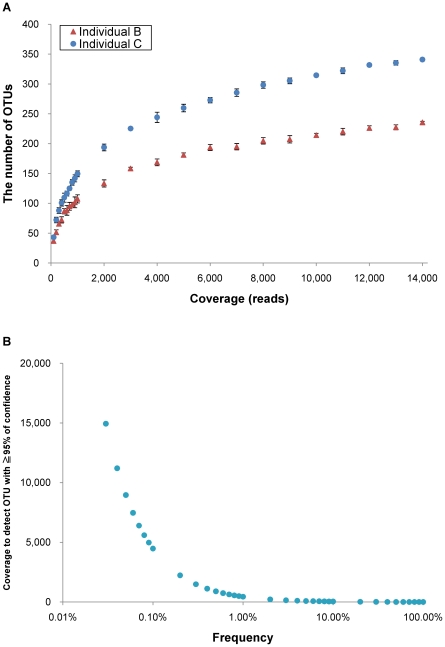
Association between coverage and the number of OTUs. (A) The number of OTUs sampled as a function of number of reads. The data points represent mean ± SD of five randomized samplings. (B) Coverage to detect OTU with different frequencies with ≥95% of confidence. The data points were estimated based on the binomial distribution (see Materials and Methods). The x axis is shown in logarithmic scale.

The number of OTUs for all reads were 240 and 346 for sample B and C, respectively. After denoise process by AmpliconNoise, the latest version of Pyronoise [10], this number became 237 and 350, respectively. The number of OTUs in two samples, B and C, increased according to coverage and did not reach a plateau, even at 14,000 reads (Figure 2A). Figure 2B shows the coverage necessary to detect a given OTU with ≥95% confidence based on the binominal distribution. In order to detect an OTU present with 0.1% of frequency, >4,000 reads are required. For an OTU with a frequency of 0.01%, >44,000 reads are required.

### Effect of different experimental procedures

**Figure 3:**
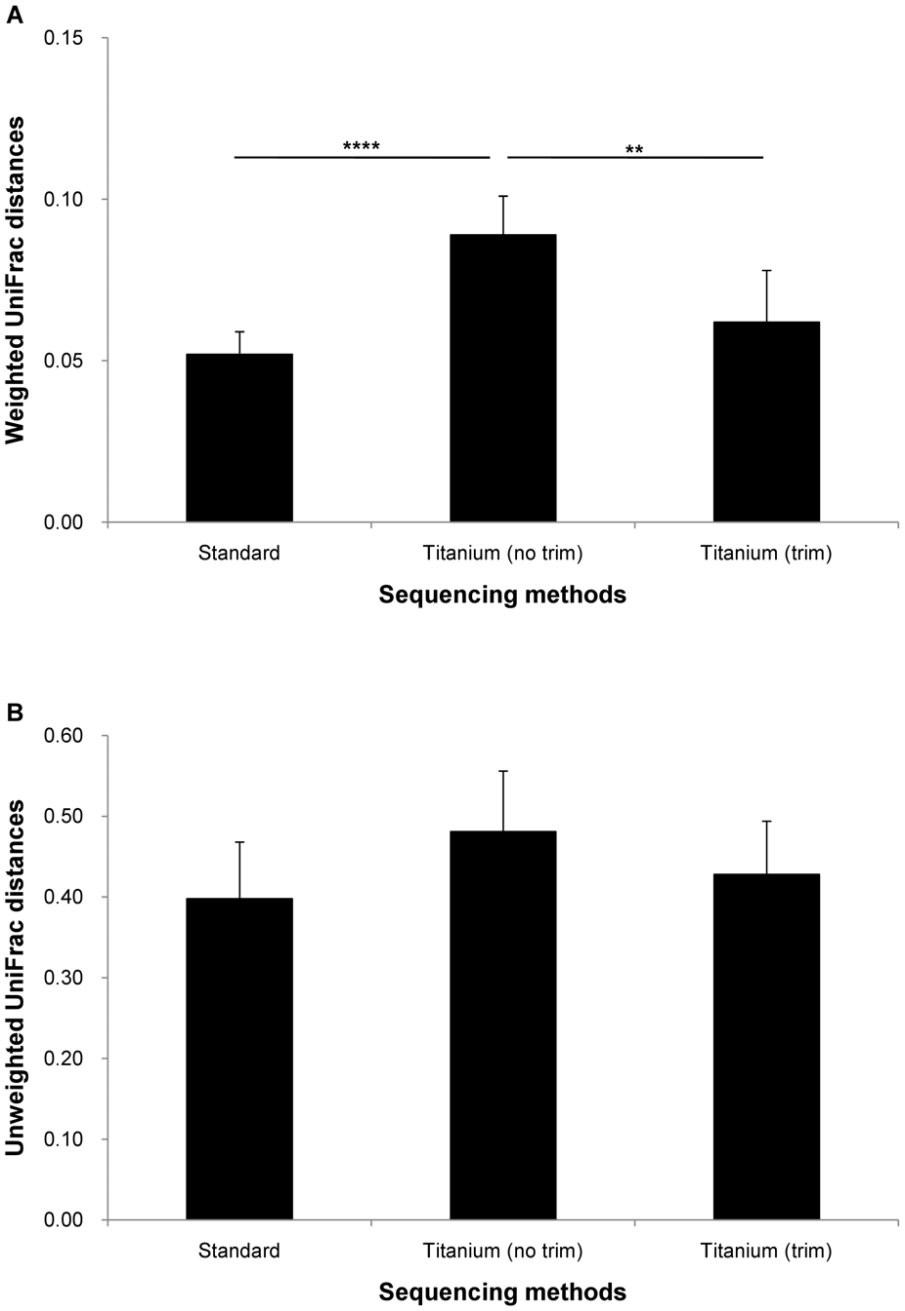
UniFrac distances between sequencing methods. (A) Weighted and (B) unweighted UniFrac distances. Reads by 454 Standard chemistry were compared with technical replicates of the same DNAs sequenced by 454 Standard chemistry (Standard), with 454 Titanium chemistry (no trim), and with 454 Titanium chemistry with sequences trimmed at 270 bp (trim). ** p<0.01, **** p<0.0001

Figure 3 shows both UniFrac distances comparing reads by 454 Standard chemistry versus those of the same DNAs by 454 Standard chemistry (technical replicates) and by 454 Titanium chemistry. Significant difference (t_10_ = 6.45, p = 7.33×10^−5^) in weighted UniFrac distances was observed between both chemistries. Since this difference was likely due to difference in length of reads (mean ± SD, Standard: 269.4±9.8 bp, Titanium: 329.2±11.8 bp), the Titanium reads were trimmed to 270 bp in order to correct for this effect. Difference in weighted UniFrac distance became not significant (t_10_ = 1.38, p = 0.199). Unweighted UniFrac distances were not different between the three sequence comparisons (Figure 3B).

**Figure 4 pone-0016952-g004:**
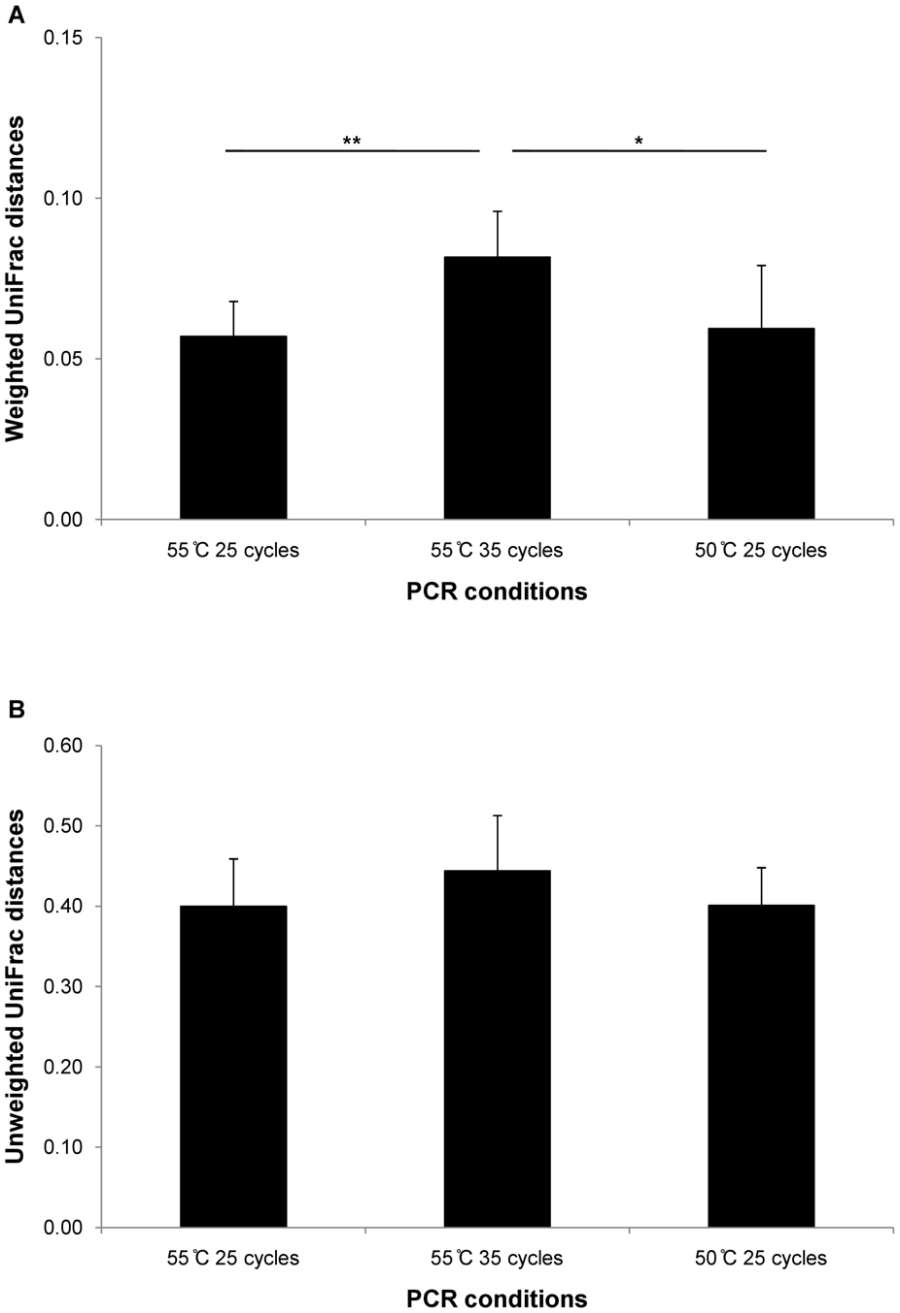
UniFrac distances between different PCR conditions. (A) Weighted and (B) unweighted UniFrac distances. UniFrac distances were calculated between the most used PCR condition (annealing temperature: 55°C and the number of cycle: 25 cycles) and three PCR conditions; the same PCR condition (technical replication), the PCR condition of more cycles (55°C and 35 cycles), and the PCR condition of lower annealing temperature (50°C and 25 cycles). * p<0.05, ** p<0.01

Figure 4 shows both UniFrac distances between the most used PCR condition in this study (annealing temperature 55°C and 25 cycles) and three PCR conditions. The PCR condition (55°C and 25 cycles) was compared to a technical replicate, the PCR condition of more cycles (55°C and 35 cycles), and the PCR condition of lower annealing temperature (50°C and 25 cycles). We observed differences in weighted UniFrac distances between 55°C /25 cycles and 55°C /35 cycles (t_12_ = 3.68, p = 3.13×10^−3^) and between 55°C /35 cycles and 50°C /25 cycles (t_11_ = 2.30, p = 0.042).

**Figure 5:**
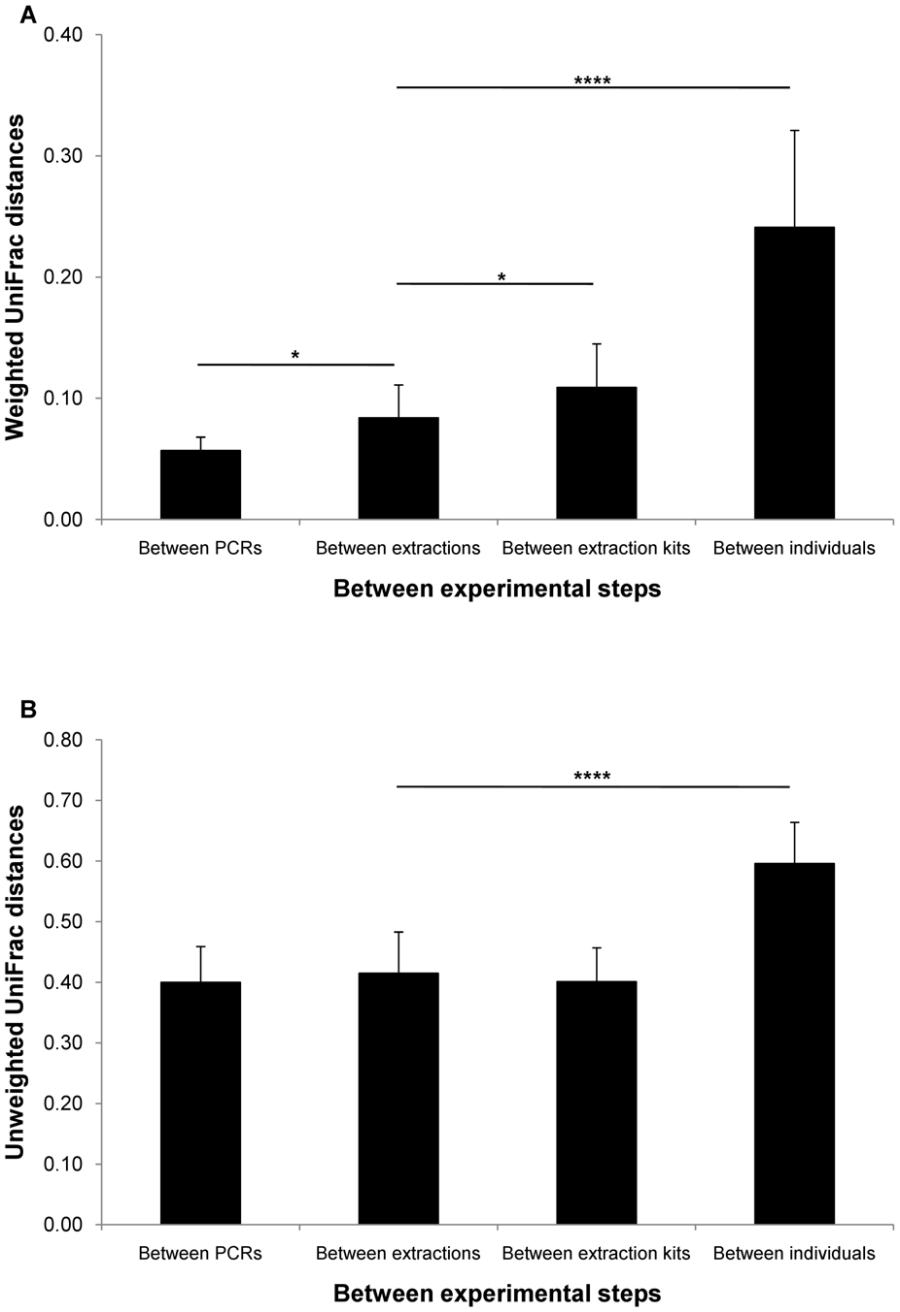
Effect of different experimental steps measured by UniFrac distances. (A) Weighted and (B) unweighted UniFrac distances. ‘Between extractions’ was used as a base measurement for comparison with other distances. Between PCRs: distances between PCRs using the same DNAs (n = 8). Between extractions: distances between extractions of DNAs from two biopsy specimens from the same anatomic region or the same stool specimens by the Stool kit (n = 18). Between extraction kits: distances between extractions of DNAs from two biopsy specimens from the same anatomic region by the Stool kit and the Mini kit (n = 12). Between individuals: distances between 2 of 9 individuals from biopsies from transverse colon. Mean ± SD, * p<0.05, ****: p<0.0001

Figure 5 shows both UniFrac distances between different experimental procedures. We used ‘Between extractions’ as a base measurement for comparison with other procedure steps. ‘Between extractions’ was obtained from two different specimens from the same anatomic region or the same stool specimens. Comparing the distance measurement of “Between extractions” with those of “Between extraction kits” and “Between individuals” allowed us to estimate the magnitude of the effects of ‘Between extraction kits’ and ‘Between individuals’. In all of the comparisons, differences between individuals were much larger quantitatively (t_52_ = 8.13, p = 7.94×10^−11^) and qualitatively (t_52_ = 9.26, p = 1.39×10^−12^) than any other effects. Even if we eliminated an ulcerative colitis and a Crohn’s disease patient, both UniFrac distances were not changed (data not shown). We also observed other significant differences in weighted UniFrac distance at “Between PCRs” (t_24_ = 2.72, p = 0.012) and at “Between extraction kits” (t_28_ = 2.18, p = 0.038).

We compared OTUs at the phylum level to find out what contributed to the quantitative differences between extraction kits; QIAamp DNA Stool Mini Kit (QIAGEN, Amsterdam, The Netherlands), referred in what follows as Stool kit and QIAamp DNA Mini Kit (QIAGEN), referred to in what follows as Mini kit. The two extraction kits produced very different proportions of *Bacteroidetes*. The proportion of *Bacteroidetes* extracted by the Stool kit was significantly higher than the Mini kit (paired t test: t_11_ = −5.94, p = 9.69×10^−5^). 11 of 12 replicates showed a higher proportion of *Bacteroidetes* using the Stool kit and differences were from −0.5% to 18.3% (mean ± SD: 10.1±5.9%). Table 1 shows the top 4 classes which occupied > 88.0% of all reads. All proportions were changed but *Firmicutes Bacilli* was barely extracted by the Mini kit.

**Table 1:** Proportion of the top 4 bacterial classes in the six biopsies of two individuals by different extraction kits (Mini Kit and Stool Kit).

Individual ID	Locus	*Bacilli*		*Bacteroidetes*		*Clostridia*		*Verrucomicrobiae*	
		Mini	Stool	Mini	Stool	Mini	Stool	Mini	Stool
C	cecum	0.0%	3.3%	51.5%	58.8%	45.5%	35.8%	0.5%	0.3%
C	ascending colon	0.0%	4.8%	45.8%	54.3%	49.0%	37.0%	0.5%	0.5%
C	transverse colon	0.0%	2.3%	46.5%	56.8%	51.3%	39.0%	1.3%	0.3%
C	descending colon	0.0%	4.8%	48.8%	50.1%	48.0%	41.1%	1.3%	1.0%
C	sigmoid colon	0.3%	1.8%	50.5%	49.8%	47.0%	44.3%	0.3%	1.0%
C	rectum	0.0%	1.8%	46.8%	52.0%	49.0%	43.5%	0.3%	0.5%
D	cecum	0.0%	8.0%	40.8%	53.0%	36.0%	28.8%	12.0%	6.0%
D	ascending colon	0.0%	4.5%	36.0%	54.3%	38.8%	33.3%	15.8%	4.5%
D	transverse colon	0.0%	4.8%	38.8%	52.5%	38.3%	32.8%	13.3%	5.3%
D	descending colon	0.3%	3.8%	39.5%	54.8%	40.8%	30.5%	11.5%	6.3%
D	sigmoid colon	0.0%	5.5%	36.3%	52.8%	44.0%	30.0%	9.8%	4.5%
D	rectum	0.0%	4.0%	41.0%	52.5%	40.0%	28.5%	10.3%	7.3%

### Differences between anatomic regions

Table 2 shows the average of both UniFrac distances between the biopsies of the seven anatomical regions and two sampling types of stools. Significance was tested with each UniFrac distance with that of two extractions from the same anatomic region (weighted: 0.084±0.027, unweighted: 0.415±0.068). No quantitative and qualitative significant differences were observed within colon from ileum to rectum, but there was a significant positive trend between distances within colon and weighted UniFrac distances (permutation test: p<0.011). Each individual shows the same tendency, although the degree was very different in each individual (both sides test: p = 4.02×10^−4^, 0.012, 0.115, 0.309). A significant difference was mostly observed in weighted UniFrac distances between biopsies from colon and colonoscopy stool (t test: p = 0.300∼3.58×10^−3^) and between biopsies from colon and fresh stool (p = 1.12×10^−3^∼9.42×10^−5^). Significant differences in unweighted UniFrac distances were only found between biopsies of colon and fresh stool (p = 0.069∼0.011).

**Table 2:** UniFrac distances between the seven biopsies and two sampling types of stools.

	ileum		cecum		ascending colon		transverse colon		descending colon		sigmoid colon		rectum		colonoscopy stool		fresh stool	
ileum	###		NA		NA		0.409		NA		NA		0.402		NA		NA	
cecum	NA		###		0.350		0.380		0.389		0.362		0.426		0.449		0.516	*
ascending colon	NA		0.066		###		0.403		0.398		0.392		0.442		0.474		0.499	*
transverse colon	0.067		0.098		0.078		###		0.384		0.389		0.394		0.426		0.489	*
descending colon	NA		0.100		0.070		0.064		###		0.368		0.418		0.464		0.510	*
sigmoid colon	NA		0.134		0.103		0.083		0.072		###		0.410		0.433		0.484	
rectum	0.074		0.144		0.125		0.080		0.101		0.080		###		0.474		0.505	*
colonoscopy stool	NA		0.181	**	0.159	*	0.144	*	0.130	*	0.112		0.100		###		0.459	
fresh stool	NA		0.237	****	0.208	***	0.189	***	0.182	***	0.159	**	0.154	***	0.146	**	###	

Weighted distances are shown in the cells below the diagonal and unweighted distances are shown above the diagonal. Significance was tested with each UniFrac distance between that of two extractions from the same anatomic region (Mean ± SD, weighted: 0.084±0.027, unweighted: 0.415±0.068).

* p<0.05,

** p<0.01,

*** p<0.001,

**** p<0.0001. Significant p values after Bonferroni correction ( =  1.67×10^−3^) are underlined.

## Discussion

UniFrac distances between and within samples decreased (Figure 1A and 1B) from 0 to 1,000 reads. These data suggest that at least 1,000 reads are required to sample the majority of bacteria contributing to quantitative and qualitative UniFrac distances in human colon samples. This finding provides important observations for future experiments. (1) All distances decreased according to coverage up to 1,000 reads. However, if samples have less than 1,000 reads comparisons should be made by standardizing the number of reads to a common number to draw valid conclusions as in this study. (2) All distances were stable after 1,000 reads. In order to investigate association between samples using both UniFrac distances very little would be gained by analyzing more than 1,000 reads. (3) Even a very small number of reads, for instance fewer than 100, can distinguish individuals as shown before [5]. On the other hand, this would vary even within the same individual, depending on the sample source, because of diversity of bacterial content [7].

Erroneous inflation of OTUs as shown in [10] was not observed. This would be caused by different methods to assign an OTU to each sequence read. Quince *et al*. [10] assigned different OTUs to two sequence reads if their differences were more than a threshold. Thus, the number of OTUs is sensitive to sequencing error of 454. In contrast, we assigned one of known OTUs to each sequence read. Even if there are noises of 454 in our method, it is less likely to assign another OTU instead of the same OTU, which did not lead to erroneous inflation of OTUs. The number of OTUs increased when the number of reads increased and it did not reach a plateau even at 14,000 reads as shown in Figure 2A. This trend has been observed by other investigators (e.g. [4], [7]). However, it is difficult to judge how much the number reads should be increased to analyze rare bacteria because a rare OTU needs a very large number of reads. For instance, OTU with 0.01% of frequency required >44,000 reads/sample to stably analyze their presence or absence in a given sample (Figure 2B). Additionally a deeper classification is difficult to pursue, analyzing the presence or absence of a specific bacteria without phylogenic tree. In order to analyze very rare bacteria, researchers need to use target-specific primer sets (e.g., [11]), instead of the consensus primer sets for the broadly conserved bacterial sequences.

Significant inflation of unweighted UniFrac distances caused by chimeric sequences was observed between individuals. Difference in this distances were relatively small and would not influence conclusion about difference between individuals as discussed before [6]. However, elimination of chimeric DNAs should be done to analyze smaller differences.

Taken all together, it would be reasonable to analyze 1,000 reads for all samples as recommended before [5]. However, due to the variability of number of reads in different samples, it is important to adjust all samples to a common number of reads, ideally to at least 1000 reads, since both UniFrac distances were highly influenced by the number of reads.

Differences between individuals were much higher than any other differences (Figure 3–5). However, at class level, all top 4 classes of bacteria changed their proportion between the two extraction methods. Recently, Wu *et al*. [12] also found some extraction methods influence the proportion of *Firmicutes*. A decrease of *Firmicutes Bacilli* would be a direct effect of the low extraction efficiency with the Mini kit. This primary effect was likely due to the 95°C of 5 min incubation that was included in the Stool kit and omitted in the Mini kit. This finding supports the Wu *et al* [12]'s assumption at least for *Firmicutes Bacilli* that ‘the recovery of *Firmicutes* was increased while the recovery of *Bacteriodetes* was decreased, possibly a result of improved lysis of *Firmicutes* with these methods’.

Quantitative and qualitative analysis using UniFrac distances showed that difference from ileum to rectum was similar to that between two different biopsies from the same anatomic region (Table 2). This suggested that the majority of bacteria would be similar throughout ileum to rectum. However, there was a significant positive trend between distances within colon and weighted UniFrac distances. This tendency was conserved in each individual. However, its degree seemed very different. Future studies are needed to reveal if there are individual differences in diversity of microbes. On the other hand, significant differences without Bonferroni correction were observed in similar comparisons; between biopsies and colonoscopy stool for weighted distances, between biopsies and fresh stool for weighted distances, and between biopsies and fresh stool for unweighted distances. Thus, we concluded that bacteria from biopsies in the intestine were quantitatively different from the stool under colonoscopy and quantitatively and qualitatively different from the fresh stool. This is consistent with results by Sanger sequencing colonies of amplicons [8]. Since stools aspirated under colonoscopy and fresh stools are generally considered a mixture of bacteria from upper organs and colon, it would obscure weak associations between bacteria and host genetic factors. Taken all together, for a systematic sampling and analysis of bacterial content, at least one biopsy from ileum to rectum, not stool, should be included as representative of colon bacteria.

Bacteria analyzed in this study are attached to biopsy after bowel cleansing. Bowel cleansing is most probably able to impact on the luminal flora than the adherent flora sampled by biopsy. It would be practically difficult to assess the extent of that influence in human samples. However, our results showed positive trends between quantitative distances and anatomic distances and differences between biopsies and stools under colonoscopy. These results suggested that influence of bowel cleansing was not large enough to diminish these differences.

Recently, Kuczynski *et al*. [13] compared microbial community resemblance methods and revealed differences in their abilities to detect some biological relevant patterns. From this point of view, the method used for the present work (UniFrac) was effective in revealing not only individual differences, but also other differences caused by experimental conditions (Figure 3–5). Differences caused by different extractions of biopsies from the same intestinal site were also significantly detected, although the differences were very small.

In conclusion, results of human colonic bacteria using high-throughput sequencing were highly dependent on the experimental design, especially the number of sequence reads, the extraction method, and the sample type. We recommend that for future research (1) at least 1,000 reads are analyzed for all samples (2) that the same experimental methods are used for all the samples, (3) and that one biopsy specimen from ileum to rectum, not stool, is collected for a systematic sampling and analysis of human colon bacteria by high-throughput methods.

## Materials and Methods

### Ethics Statement

The protocol was reviewed and approved by the Ethical Committee of the University of Liège and every patient signed a written informed consent.

### Subjects

Nine individuals of Western European descents (labeled A to I) were sampled, four males and five females between 25 and 62 years of age. Sample B was an ulcerative colitis patient and Sample D was a Crohn's disease patient. The other individuals were from subjects undergoing colonoscopy as a screening procedure for colo-rectal cancer.

### Sampling

Biopsies from six anatomic regions in colon (cecum, ascending colon, transverse colon, descending colon, sigmoid colon, and rectum), stool aspirated during colonoscopy, and fresh stool were collected from individuals A–D. Just before starting the colonoscopy preparations, they collected fresh stools into a sterile plastic container. Within four hours after sampling, fresh stools were stored in −80°C until extraction of DNA. Biopsies were also collected from three anatomic regions (ileum, transverse colon, rectum) from individuals E–I. Prior to the collection of biopsy specimens, the nine individuals received a standard bowel cleaning with a polyethylene glycol preparation using Moviprep (Norgine, Uxbridge, UK). When the biopsy was grasped with the forceps, it was trapped inside the forceps without direct contact with the inner operating channel of the endoscope. The forceps used was carefully washed between each biopsy and was changed for every patient. Therefore, the risk of contamination is minimal. Biopsies were immediately frozen in liquid nitrogen, and stored at −80°C. During colonoscopy, liquid stools were aspirated in different segments including rectum, and collected. The liquid stool samples were centrifuged at 5,000 rpm for 5 min and the precipitate was stored at −80°C until further analysis.

### DNA extractions

DNA was extracted from stool specimens using the Stool kit according to manufacturer's protocol. Stool specimens were disrupted and homogenized in buffer ASL of the Stool kit with 5 mm stainless steel beads using a Tissue lyser (Qiagen) at a rate of 25 times/sec for 2 min, followed by incubation at 95°C for 5 min. The Stool kit was also used for DNA extraction from the biopsy specimens. For comparison (12 pairs), we also used the Mini kit. The primary difference between the two extraction protocols was the inclusion of a 5 min 95°C incubation in ASL buffer in the Stool kit, which according to the manufacturer has an increased effect in lysing bacteria. Biopsy specimens were also disrupted and homogenized following the same procedures as the stool specimens. The extractions were done following the manufacturer's instructions except that the proteinase K reaction was carried out at 70°C as in the Stool kit. Eighteen extractions were replicated using two specimens from the same anatomical region (11 pairs) or stools (7 pairs). Every extraction was carried out without specimens as negative control, and we confirmed no amplification of the negative control in the PCR step. Extracted DNAs were stored at −20°C until used.

### PCR amplification and sequencing of the V2 region of bacterial 16S rRNA

The V2 region of 16S rRNA was amplified using a primer set reported by Hamady *et al.* [14]. For 454 Standard chemistry, the forward primer (5′-GCCTTGCCAGCCCGCTCAG *TC*AGAGTTTGATCCTGGCTCAG-3′) contained the primer B sequence used in the 454 Genome Sequencer FLX instrument (Roche, Basel, Switzerland), a two-base linker sequence (TC), and the broadly conserved bacterial primer. The reverse primers (5′-GCCTCCCTCGCGCCATCAG NNNNNNNN*CA*TGCTGCCTCCCGTAGGAGT-3′) contained primer A, eight base bar codes (Ns) to distinguish samples, a two-base linker sequence (CA), and the broadly conserved bacterial primer. For 454 Titanium chemistry, we used CCATCTCATCCCTGCGTGTCTCCGACTCAG and CCTATCCCCTGTGTGCCTTGGCAGTCTCAG as adapter sequence A and B, respectively.

A 30 µl PCR reaction contained 6 µl of 5x Phusion HF buffer, 200 µM each of dNTP, 0.5 µM of each primer pair, and 0.6 U of Phusion High-Fidelity DNA Polymerase (Finnzymes OY, Espoom, Finland). The PCR conditions in most experiment were 98°C for 2 min, 25 cycles at 98°C for 30 sec, 55°C for 30 sec and 72°C for 30 sec, followed by 72°C for 10 min on a GeneAmp PCR System 2700 thermal cycler (Applied Biosystems, Foster City, CA). As comparison, we used two different PCR conditions. Annealing temperature was changed to 50°C and the number of cycles was changed to 35 cycles.

Amplicons were purified using MultiScreen PCR_µ96_ Filter Plate (Millipore, Billerica, MA). DNA concentrations were measured by the Quant-iT PicoGreen dsDNA reagent and kit (Invitrogen, Carlsbad, CA). Based on DNA concentration, amplicons were combined in equimolar ratios. Pooled amplicons were treated by Montage PCR Filter Units (Millipore) to concentrate amplicons and AMPure kit (Agencourt Bioscience, Beverly, MA) to remove amplicons shorter than 100 bp. The final concentration and distribution of length were measured using the Experion DNA 1K Analysis kit (Bio-Rad, Hercules, CA) on Experion Automated Electrophoresis Station (Bio-Rad). Pyrosequencing was carried out using primer A on a 454 Genome Sequencer FLX instrument [15].

### Sequence analysis

Image processing and data processing for amplicon sequencing were done using the Genome Sequencer FLX System Software Package 2.3 (Roche). From sff files, sequences passing the following criteria were assigned to each sample using custom-made scripts; perfectly identical sequences with the bar code and the primer sequence, length ≥200 bp, averaged quality score ≥25, and no ambiguous characters. Definite chimeric sequences detected by B2C2 software were eliminated [9].

OTU was assigned to each read using the best Megablast hit [16] against Greengenes core set of May 2009 [17] using the following settings; e value > 1×10^−15^, identity > 90%, and word size = 42. OTUs were assigned to 99.95% of reads and the remaining reads were excluded from further study.

To determine difference between two given samples, we used the UniFrac distances with the reference tree of the Greengenes core set as described before [18]. In brief, UniFrac distance between two given samples was calculated as the ratio of the length of all sample specific branches over the total branch length, after the reads from the two samples were assigned to a phylogenic tree. While normalized weighted UniFrac distance uses abundance of each OTU as a quantitative value, unweighted UniFrac distance uses only presence or absence of each OTU as a qualitative value [19]. Both UniFrac distance values are between 0 and 1. A smaller distance means that the two given samples shared more bacteria.

### Coverage and UniFrac distances

To reveal associations between coverage and weighted and unweighted UniFrac distances we sequenced two times the 16S rRNA from ascending colon of two individuals (Samples B and C). The number of reads were 7,360 for sample B-1, 8,128 for B-2, 7,122 for C-1, and 7,703 for C-2. From each sample read data set, different numbers of reads were randomly sampled three times at increments of 20 reads from 20 to 100 reads, at increments of 200 reads from 200 to 1,000 reads, and at increments of 1,000 reads from 2,000 to 7,000 reads. Both UniFrac distances were calculated between the same number of reads from different read data of the same individuals (B-1 and B-2, C-1 and C-2) and between the two individuals (B-1 and C-1, B-1 and C-2, B-2 and C-1, B-2 and C-2).

### Coverage and the number of OTUs

The two deep sequencing data sets (B-1 and B-2, C-1 and C-2) were merged for each individual and randomly sampled for different number of reads five times at increment of 100 reads from 100 to 1,000 reads, and at increments of 1,000 reads from 1,000 to 14,000 reads. For each random dataset, the number of OTUs was obtained.

### Frequency and required coverage

One of the motivations to use high-throughput sequencing is to detect specific bacteria associated with diseases and phenotypes. It is important to analyze a high enough coverage to be able to detect such bacteria stably, since rare bacteria require deeper coverage. The coverage required to detect a given OTU with 95% confidence was estimated based on the binominal distribution.
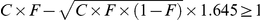



where C is the coverage and F the frequency of a given OTU. The lower z-score at a 90% confidence interval is −1.645. Using this calculation, association between frequency and the required coverage was examined.

### Effect of different experimental procedures

To validate our conclusions on the different experimental procedures independent of sequence coverage comparisons were made at the same number of reads, i.e. 400 reads for each sample. Differences between 454 Standard and Titanium chemistries of the same DNA samples (n = 6), between PCR conditions (n = 7), between extractions (n = 18) and between the two DNA extraction methods (n = 12), were analyzed by UniFrac distances and proportion of bacteria at phylum and class levels.

### Difference between anatomic regions and stools

As mentioned above, 400 sequence reads were used to calculate both UniFrac distances between the seven anatomic regions and the two stools (stools under colonoscopy and fresh stools). Significance was tested with each UniFrac distance between that of two extractions from the same anatomic region. In order to investigate if UniFrac distances increase as anatomic distances between loci increase, we performed a permutation test on the results of Table 2. Based on Table 2, we compared 23 adjacent distances for each UniFrac distance, respectively; for instance, cecum/ascending colon and cecum/transverse colon; cecum/transverse colon and cecum/descending colon. We counted the number of comparisons which showed more UniFrac distances when anatomic distances increased. The statistical significance of this number was assessed by permutation of all possibilities with both sides test. The same analysis was possible for each individual A–D who provided biopsies from six anatomic regions (20 comparisons).

### Statistical analysis

Student t-test and paired t-test were used for comparison. Statistical analysis was done using R 2.8.1 (http://www.R-project.org). Raw p values are shown without any correction because one of purposes of this paper was to show potential differences caused by different experimental steps.
